# Identifying sex similarities and differences in structure and function of the sinoatrial node in the mouse heart

**DOI:** 10.3389/fmed.2024.1488478

**Published:** 2024-12-05

**Authors:** Zeyuan Yin, Eleonora Torre, Manon Marrot, Colin H. Peters, Amy Feather, William G. Nichols, Sunil Jit R. J. Logantha, Areej Arshad, Simran Agnes Martis, Nilay Tugba Ozturk, Weixuan Chen, Jiaxuan Liu, Jingmo Qu, Min Zi, Elizabeth J. Cartwright, Catherine Proenza, Angelo Torrente, Matteo E. Mangoni, Halina Dobrzynski, Andrew J. Atkinson

**Affiliations:** ^1^Division of Cardiovascular Sciences, School of Medical Sciences, University of Manchester, Manchester, United Kingdom; ^2^Institut de Génomique Fonctionnelle, Université de Montpellier CNRS, INSERM, Montpellier, France; ^3^Laboratory of Excellence Ion Channels Science and Therapeutics (ICST), Valbonne, France; ^4^Department of Physiology and Biophysics, University of Colorado Anschutz Medical Campus, Aurora, CO, United States; ^5^Department of Cardiovascular and Metabolic Medicine and Liverpool Centre for Cardiovascular Science, University of Liverpool, Liverpool, United Kingdom; ^6^Department of Anatomy, Jagiellonian University Medical College, Kraków, Poland

**Keywords:** heart rate, sex, sinoatrial node, diastolic depolarization, electrophysiology, ion channels

## Abstract

**Background:**

The sinoatrial node (SN) generates the heart rate (HR). Its spontaneous activity is regulated by a complex interplay between the modulation by the autonomic nervous system (ANS) and intrinsic factors including ion channels in SN cells. However, the systemic and intrinsic regulatory mechanisms are still poorly understood. This study aimed to elucidate the sex-specific differences in heart morphology and SN function, particularly focusing on basal HR, expression and function of hyperpolarization-activated HCN4 and HCN1 channels and mRNA abundance of ion channels and mRNA abundance of ion channels contributing to diastolic depolarization (DD) and spontaneous action potentials (APs).

**Methods:**

Body weight, heart weight and tibia length of 2- to 3-month-old male and female mice were measured. Conscious *in-vivo* HR of male and female mice was recorded via electrocardiography (ECG). Unconscious *ex-vivo* HR, stroke volume (SV) and ejection fraction (EF) were recorded via echocardiography. *Ex-vivo* HR was measured via Langendorff apparatus. Volume of atria, ventricles and whole hearts were measured from the *ex-vivo* hearts by microcomputed tomography (micro-CT). Immunohistochemistry targeting HCN4 and HCN1 was conducted in the SN and RA tissues from both male and female hearts. The funny current (*I*_f_) of SN cells in 1 nM and following wash-on of 1 μM isoproterenol (ISO) were recorded via whole cell patch clamp. The APs of SN tissue were recorded via sharp microelectrode and optical mapping of membrane voltage. The relative abundance of mRNAs was measured in male and female mice by qPCR.

**Results:**

Heart weight to tibia length ratio and heart volume of females were significantly smaller than males. Unconscious *in-vivo* HR in male mice was higher than that in females. Conscious *in-vivo* HR, *ex-vivo* HR, SV, and EF showed no notable difference between male and female mice. Immunohistochemistry revealed HCN4, HCN1, and the sum of HCN4 and HCN1, expression in the SN was notably elevated compared with the RA in both male and females, but there was no sex difference in these channels expression. There were also no significant sex differences in the *V*_0.5_ of *I*_f_ in SN cells in the presence of 1 nM ISO, however wash-on 1 μM ISO in the same cells induced a significantly increased shift of *V*_0.5_ to more positive voltages in males than in females. The expression of mRNA coding for adrenergic receptor beta-1 (Adrb1) and cholinergic receptors muscarinic 2 (chrm2) in male mice was higher compared with that in female mice. Early diastolic depolarization (EDD) rate in APs from peripheral SN (pSN) from male mice were higher than these in female mice. Mice of both sexes showed equivalent frequency of SN APs and spatial localization of the leading site in control, and similar significant response to ISO 100 nM superfusion.

**Conclusion:**

Males display faster *in-vivo* HR, but not *ex-vivo* HR, than females associated with increased expression of Adrb1 in male versus female. This suggests a possible difference in the β-adrenergic modulation in males and females, possibly related to the greater ISO response of *I*_f_ observed in cells from males. The role of hormonal influences or differential expression of other ion channels may explain these sex-specific variations in HR dynamics. Further investigations are necessary to pinpoint the precise molecular substrates responsible for these differences.

## 1 Introduction

Heart automaticity and contractility sustain everyday life with remarkable robustness and flexibility. The cardiac conduction system (CCS) initiates and coordinates the automatic electrical impulses that trigger heartbeats, ensuring synchronized contraction of the heart muscle (1). The CCS system includes sinoatrial node (SN), atrioventricular node (AVN), His bundle and its branches and Purkinje network (2). The CCS ensures the sequence of contractions from the atria to the ventricles, thereby enabling effective circulation of blood throughout the body (3, 4). The SN is the heart’s primary pacemaker centre within the CCS under physiologic conditions (5–7) and generates the primary pacemaker impulse. SN pacemaker activity is due to diastolic depolarization (DD), a slow depolarising phase of the AP which leads the membrane voltage, from the end of repolarization phase of the preceding action potential to the threshold of the following AP (8, 9).

Several ion channels of the plasma membrane of SN pacemaker cells contribute to DD (8). Among those, the hyperpolarization activated “funny” current (*I*_f_) plays a major role in the generation of DD (9). *I*_f_ is constitutively activated at a low fractional activation throughout the cardiac cycle and contributes a substantial fraction of the net inward current during diastole to generate spontaneous activity in SN cells (9–11). Hyperpolarization-activated cyclic nucleotide-gated channels 1 and 4 (HCN1 and HCN4) encode the SN *I*_f_, although the isoform expression levels differ in different species (12). The open probability of HCN4 channels is positively modulated by variations in intracellular cyclic AMP (cAMP) (13). This cAMP-mediated mechanism is important in modulation of heart rate (HR) by the autonomic nervous system (ANS) (14). More generally, the degree of expression and gating of HCN channels influence HR. These properties include voltage dependence, current amplitude and gating kinetics (15, 16). Beside HCN channels, voltage-gated L-type Ca_V_1.3 (17), T-type Ca_V_3.1 (18) Ca^2+^ channels and activity of the Na^+^–Ca^2+^ exchanger (NCX1) are involved in the generation of DD (19).

Sex differences have been observed in HR and heart rate variability (HRV) across various species, including humans, rodents, and other animals (20). In humans, males tend to exhibit greater increases in HR in response to physical exercise with quicker recovery times (21, 22). Males typically show higher HRV, suggesting greater autonomic regulation, with these differences diminishing with age (23). In rodents, such as rats and mice, males show more pronounced HR increases in response to stress (22, 24, 25). Male rodents tend to have higher HRV, indicating robust autonomic control, which can be influenced by the oestrous cycle in females. In other species, like dogs, males usually have higher HRV compared to females, while studies in horses present mixed results regarding sex differences in HR and HRV (26, 27). Generally, across species, males display higher HRV, reflecting stronger autonomic regulation and a more significant HR response to stress and exercise. These differences are influenced by hormonal, physiological, and behavioural factors, which vary across species and change with age (20, 27). Sex hormones, notably oestrogen and progesterone, alongside variations in beta-adrenergic responses, contribute to these observed sex-based distinctions in HR and its associated physiology (28–30). Our previous work has revealed significant differences in key components of the SN pacemaking mechanism between the sexes in rats. Notably, mRNA levels and protein expression of Ca_V_1.3 channels which underlie the diastolic component of *I*_Ca,L_, KCNJ3 (K_ir_3.1) which contributes to G protein-activated inwardly rectifying potassium current (*I*_KACh_), and NKX2-5 which is a key transcriptional factor inhibiting the development of SN myocytes cell fate, were found to be higher in the female SN (31). These sex-specific differences in HR regulation are vital considerations in development of personalized medicine (32, 33).

This study sets out to elucidate sex-specific differences in the electrophysiological characteristics of the SN in mice. We focus on differences in HR, SN properties, spontaneous AP, *I*_f_ and SN HCN channel isoforms, HCN4.

## 2 Materials and methods

### 2.1 Animals information

All animal experiments apart from patch clamp and optical mapping were performed in the UK in compliance with the University of Manchester and UK Animals (Scientific Procedures) Act (1986), the European directive 2010/63/EU, the ethical panel of University of Montpellier and by The French Ministry of Agriculture (Protocol No. 2017010310594939). Feeding, culling mice, dissection of SN tissue, dissociation and patch clamp of SN cells were performed in accordance with the US Animal Welfare Act and was carried out according to a protocol approved by the University of Colorado Anschutz Medical Campus Institutional Animal Care and Use Committee. 2- to 3-month-old C57BL6J male or female mice (Jackson Labs) were used, with animals weighing 16.85 to 20.6 g (female) and 23.27 to 30.5 g (male).

### 2.2 Phenotypic measurements

Body weight, heart weight and tibia length measurements were taken for each mouse (*N* = 13 for each group).

### 2.3 Micro-CT scans of mouse hearts

Eight mice (*N* = 4 for females and *N* = 4 for males) were culled via cervical dislocation and the hearts collected. The hearts were immersed in a 7.5% I2KI for 24 h. Micro-CT scanning was conducted utilizing a Nikon Metris XTEK high flux bay 225 V scanner at the Henry Moseley Manchester X-ray Imaging Facility, University of Manchester. The hearts were immobilized in a sealed container to prevent tissue movement and shrinkage. 360° scans using energy levels of 85–180 kV were performed. Amira 6.5.0 software (Thermo Fisher) was used for all scan data analysis for micro-CT imaging following established methods (34–36). The SN, atria and ventricles were segmented according to anatomical landmarks and image intensity differences.

### 2.4 Electrocardiography recording of conscious mice

Conscious electrocardiography (ECG) recordings were performed on the mice (*N* = 6 for males; *N* = 7 for females) using the ECGenie device (Mouse Specifics Inc.), which uses footpad ECG electrodes to non-invasively record the ECG. The signals were amplified through an Animal Bioamp connected to a Powerlab amplifier (ADInstruments). Each mouse was acclimatized to the setup for 10 min before data collection. Only data from continuous recordings were used in the analysis. Subsequent offline analysis of the ECGs was conducted using Labchart v8 (ADInstruments). A peak detection algorithm within LabChart enabled R-wave identification, and the HR was calculated based on RR intervals (the interval between successive R-wave peaks). The HR was expressed as beat per minute (BPM).

### 2.5 Echography of anaesthetized mice

1.5% isoflurane anesthesia was used for echography recording with the mice’s HR being monitored and kept within a range of 400–500 BPM. The fur on the mice’s skin was trimmed, fully exposing the thoracic cage. Transthoracic echography performed using a Visualsonics Vevo 3100 imaging system. End-diastolic and end-systolic left ventricular volumes (LVVd/s) were assessed via imaging in the parasternal long-axis view to determine EF using the following the formula:


$$EF\%=\left(\mathrm{LVVd}-\mathrm{LVVs}\right)/\mathrm{LVVd}\times 100$$

SV is calculated using the formula:


SV=End−dialostic volume−End−systolic volume

### 2.6 *Ex-vivo* HR recordings

Langendorff perfusion was conducted in male and female mice hearts to record the ECG and acquire the *ex-vivo* HR. The mouse underwent euthanasia via cervical dislocation, followed by careful removal of the heart, which was then immersed in cool Tyrode’s solution (pH7.4, 120 mM NaCl, 4.0 mM KCl, 1.2 mM CaCl_2_, 1.3 mM MgSO_4_, 1.2 mM NaH_2_PO_4_, 25.2 mM NaHCO_3_, 11 mM glucose). The heart was cannulated through the aorta and retrogradely perfused with Tyrode solution continuously bubbled with 95% O_2_ at 37°C, causing closure of the aortic valve and subsequent filling of the coronary vessels. This step ensured the supply of nutrients and oxygen to the cardiac muscle, enabling the heart to beat post-excision. Extracellular potentials were continuously recorded using stainless steel pins attached to the RA and ventricular apex, amplified through an Animal Bioamp connected to a Powerlab amplifier (ADInstruments). LabChart version 8 software (ADInstruments) facilitated signal analysis, with adjustments made to low-and high-pass filters to minimize noise. Analysis in LabChart involved assessing the number of peaks within a selected range, with the time interval between consecutive R-peaks measured for each trace obtained from individual mice. The HR was calculated follow this formula and expressed as BPM:


$$HR=\frac{60}{RR\;\mathrm{interval}}$$

### 2.7 Cryosectioning

Heart specimens (*N* = 4 for each group) were affixed onto cork using OCT (Pioneer Research Chemicals) and rapidly frozen in isopentane cooled in liquid nitrogen to prevent muscle damage. The cryopreserved hearts were stored at −80°C until the cryosectioning process. Serial cryosectioning of the hearts was executed at −15°C, commencing from the ventral and proceeding to the dorsal view in the coronal plane with a 5° angle increment. The obtained sections had a thickness of 20 μm. The prepared sections were maintained at −80°C.

### 2.8 Immunohistochemical staining

All steps were performed at room temperature unless stated. Frozen tissue sections from *N* = 4 hearts for each group were fixed in a solution of 10% formalin for 30 min. This was followed by three 10-min washes in PBS (Sigma Aldrich) and a 30-min permeabilization process using 0.1% Triton X-100 in PBS. After permeabilization, the sections had three 10-min washes in PBS before treatment with a blocking solution containing 1% bovine serum albumin (BSA) in PBS. They were then incubated with a primary anti-HCN4 (APC-052; Alomone Labs, Israel) or HCN1 (APC-056; Alomone Labs, Israel) antibody derived from rabbit at a dilution of 1:50 in 1% BSA in PBS at 4°C overnight. Tissue sections were then washed three times with PBS before incubation with an FITC-conjugated goat anti-rabbit IgG secondary antibody (AP132F; Sigma) at a dilution of 1:100 in 1% BSA in PBS or a Cy3-conjugated goat anti-rabbit IgG secondary antibody (AP1823; Millipore) at a dilution of 1:500 in 1% BSA in PBS in the dark. Slides had three 10-min PBS washes before mounting with antifade mounting medium (H-1000; Vectorlab, CA, United States) and sealed with a nail polish. Slides were imaged using a Zeiss LSM5 microscope and PASCAL software (Zeiss).

### 2.9 Semi quantitative immunohistochemical analysis

After immunostaining the frozen tissue sections of SN tissue and its surrounding atrial muscle, analysis of HCN4 and HCN1 immunofluorescence was carried out using the Image J.^1^ While both fluorophores, FITC and Cy3, were used to visualize the staining, signal intensity measurements were conducted on the Cy3-stained sections. Semi-quantitative analysis was done by measuring the total pixel intensity of the HCN4-/HCN1 stained regions.

### 2.10 Intracellular AP recording

Isolation of SN tissue was based on previous studies (5, 37). The excised hearts from the mice were immersed in the prepared Tyrode’s solution. The SN tissue could be visualized along the crista terminalis (CT) with a prominent SN artery. The SN preparation was continuously perfused at 10 mL/min with bubbled Tyrode solution. Blebbistatin (Generon) of 5 μM was added and the tissue was allowed to stabilize for 30 min before recording. Microelectrodes were back-filled with 3 M KCl and inserted into an Ag-AgCl pellet holder (Harvard Apparatus, United Kingdom) prefilled with 3 M KCl. An Ag-AgCl disk electrode (Harvard Apparatus) served as bath ground return. The microelectrodes were connected to a headstage which connected to a GeneClamp 500 amplifier (Molecular Devices, United States) and Powerlab4 digitizer (ADInstruments). Intracellular APs were recorded at a 10 kHz sampling rate from the endocardial surface in intercaval myocardium surrounding the SN artery bifurcation and from distant pectinate myocardium. A minimum of five consecutive APs per impalement were analysed using LabChart v8 (ADInstruments). The relative parameters, including maximum diastolic depolarization (MDP), action potential duration (APD), APA, upstroke velocity (d*V*/d*t*), early diastolic depolarization rate (EDD) and late diastolic depolarization rate (LDD) were calculated. APs were recorded from spontaneously beating preparations.

### 2.11 Optical mapping

To evaluate the voltage changes in the SN tissue, the tissues was loaded by adding the voltage-sensitive indicator/dye Di-4-ANEPPS (8 μM) to the Tyrode’s solution (140 mM NaCl, 5.4 mM KCl, 5 mM HEPES, 5.5 mM glucose, 1 mM MgCl_2_ and 1.8 mM CaCl_2_, with pH adjusted to 7.4 with NaOH) for at least 45 min at room temperature (20–22°C). The dish was placed on an agitated plate during loading to maintain proper oxygenation of the SN tissue and load it uniformly. The dish was then constantly perfused at 34–36°C with Tyrode’s solution containing the contraction inhibitor, blebbistatin (6 μM). Recordings were performed by high speed optical voltage mapping (500 Hz) on a MiCAM Ultima-L complementary metal oxide semiconductor (CMOS) camera (256 × 256-pixel CMOS sensor, 17.6 × 17.6 mm, SciMedia). This camera was mounted on a THT microscope, with two objectives (1× and 1×) that generated a field of view of 17.6 × 17.6 mm. A led light system with built-in shutter (SciMedia) was used as an excitation light source for the voltage dye. The filter set included a 531/50-nm excitation filter, 580-nm dichroic mirror, and 580 long-pass emission filter. We usually limited our recording times to 20 s to avoid phototoxic effects of the dye. The depolarization rate was analyzed using dedicated software from the camera developer, BV Workbench 2.7.0 analysis software (Brainvision). Data on the site of electrical activity initiation (leading region, LR) were obtained with MatLab software (Matworks Inc., 2023b version). Briefly, to unequivocally define the position of the LR, two axes were considered. A first horizontal axis passed through the geometrical center of the SN tissue and a point equally distant from the ends of the RA and a second vertical axis perpendicular to the horizontal one and passing through the center of the SN tissue. These two vectors can therefore be used to classify LRs into four categories depending on the alpha angle value calculated between the horizontal axis and the axis passed through the LR and the center: I (0–90°), II (90–180°), III (180–360°) and IV (360–0°). Stimulation of the β-adrenergic pathway with 100 nM of ISO was based on the saturating doses reported by Glukov et al. (38).

### 2.12 Dissociation of single SN cells

For dissociation of individual SN cells, mice were anesthetized with isoflurane and euthanized under anesthesia by cervical dislocation. Hearts were excised and the SN dissected from the tissue while bathed in pre-warmed Tyrode’s solution (140 mM NaCl, 5.4 mM KCL, 1.2 mM KH_2_PO_4_, 5 mM HEPES, 5.55 mM glucose, 1 mM MgCl_2_ and 1.8 mM CaCl_2_, with pH adjusted to 7.4 with NaOH) containing 10 U/mL heparin. The dissected SN tissue was cut in to strips and suspended in 2.5 mL of low Ca^2+^/Mg^2+^ Tyrode’s solution (140 mM NaCl, 5.4 mM KCl, 1.2 mM KH_2_PO_4_, 5 mM HEPES, 18.5 mM glucose, 0.066 mM CaCl_2_, 50 mM 2-aminoethanesulfonic acid, 1 mg/mL BSA, pH adjusted to 6.9 with NaOH) for 5 min at 35°C and washed twice in the Tyrode’s solution with low Ca^2+^/Mg^2+^. Tissue was then digested with an enzymatic mixture containing collagenase Type II (1,064 U; L5004177; Worthington Biochemical, New Jersey, United States), elastase (9 U; LS002279; Worthington Biochemical, New Jersey, United States), and protease (65.2 μL of 1 mg/100 μL ddH_2_O; P463; Sigma) in low Ca^2+^/Mg^2+^ Tyrode’s solution for 30 min. Tissue was intermittently mixed using a pipette every 5 min during the incubation period. The incubated tissue was washed and incubated for a further 5 min with KB solution (100 mM K^+^-glutamic acid, 10 mM K^+^-aspartic acid, 25 mM KCl, 10 mM H_2_KPO_4_, 2 mM MgSO_4_, 20 mM 2-aminoethanesulfonic acid, 5 mM creatine, 0.5 mM EGTA, 20 mM glucose, 5 mM HEPES, 0.1% BSA, with pH adjusted to 7.2 with KOH) at 35°C with constant mixing and then kept at room temperature for 5 min. The isolated SN cells were re-acclimated in the following solution sequentially at room temperature: 75 μL NaCl/CaCl_2_ adaption solution (10 mM NaCl, 1.8 mM CaCl_2_) for 5 min; 160 μL NaCl/CaCl_2_ adaptation solution for 5 min; 390 μL BSA storage solution (1 mg/mL BSA, 140 mM NaCl, 5.4 mM KCL, 1.2 mM KH_2_PO_4_, 5 mM HEPES, 5.55 mM glucose, 1 mM MgCl_2_ and 1.8 mM CaCl_2_, pH adjusted to 7.4 with NaOH) for 4 min; 1.25 mL BSA storage solution for 4 min; 4.37 mL of BSA storage solution for 4 min. Finally, the isolated SN cells were stored at room temperature for up to 6 h before patch clamp experiments (39).

### 2.13 Recording of hyperpolarization-activated *I*_f_ current

Whole-cell voltage-clamp was used to record *I*_f_ following a previously used protocol (5). Data were acquired at 5–20 kHz and low-pass filtered at 10 kHz using an Axopatch 1D or 200B amplifier, Digidata 1322a or 1440a A/D converter, and ClampEx software (Molecular Devices). Reported data have been corrected for the calculated liquid junction potential error (−14 mV) using the calculator in ClampEx software. Fast (pipette) capacitance was compensated in all voltage-clamp recordings. Recording pipettes were pulled from borosilicate glass using a Sutter Instruments P-97 horizontal puller.

Cells were continuously perfused at 1–2 mL/min with recording Tyrode’s solution (140 mM NaCl, 5.4 mM KCl, 5 mM HEPES, 5 mM glucose, 1.0 mM MgCl_2_, 1.8 mM CaCl_2_, pH adjusted to 7.4 using NaOH) containing 1 mM BaCl_2_ to block inward rectifier channels and with 1 nM or 1 μM as indicated of the βAR agonist, ISO. Given that the original, though unmet, objective of the study was to record spontaneous APs and *I*_f_ from the same cells, 1 nM ISO was chosen as the appropriate control extracellular solution. 1 nM ISO stabilizes the AP firing rate of isolated pacemaker cells by eliminating frequent pauses in, and cessation of, AP firing. 1 nM does not change the AP firing rate from that observed during the bursts of APs that occur in the absence of ISO [no ISO: FR = 313 ± 31 AP/min, *n* = 19; 1 nM ISO: FR = 304 ± 15 AP/min, *n* = 50 (9);]. 1 nM ISO similarly has no effect on the voltage-dependence of activation of *I*_f_ (no ISO: *V*_1/2_ = −104.3 ± 1.5, *n* = 16; 1 nM ISO: *V*_1/2_ = −103.7 ± 2.1, *n* = 20) (10, 40).

Recording pipettes exhibited resistances of ~1.5–3.0 MΩ when filled with an intracellular solution composed of 135 mM potassium aspartate, 6.6 mM sodium phosphocreatine, 1 mM MgCl_2_, 1 mM CaCl_2_, 10 mM HEPES, 10 mM EGTA, and 4 mM Mg-ATP, with pH adjusted to 7.2 using KOH. Cells were held at-50 mV, and *I*_f_ was evoked by 3-s test pulses ranging from −50 to −160 mV in 10 mV increments (all potentials corrected for −14 mV liquid junction potential error). Conductance was calculated from inward currents following the equation:

$$G=I/\left(V-V_{r}\right)\text{,}$$

where *G* represents conductance, *I* is the time-dependent inward current amplitude at a specific voltage *V*, and *V*_r_ is the measured reversal potential for *I*_f_ under these conditions (−30 mV) (10). The conductance values were subsequently plotted against voltage and fitted with a Boltzmann equation to ascertain the *V*_0.5_:


$$f\left(V\right)=V_{\text{min}}+\frac{V_{\text{max}}-V_{\text{min}}}{1+e^{\frac{Z_{d}F}{RT}\left(V-V_{1/2}\right)}}$$

### 2.14 The extraction of total RNA and first strand cDNA synthesis

mRNA was extracted from the isolated SN tissue (*N* = 5 for male; *N* = 6 for female) using RecoverAll^™^ Total Nucleic Acid Isolation Kit (AM1975, Thermo Fisher, MA, United States). All steps followed the manufacturers protocol. Tissue was digested in protease solution. RNA was eluted into 20 μL RNAse free water. SuperScript VILO MasterMix (11755050, Thermo Fisher, MA, United States) was used for the reverse transcription of mRNA. 75 ng total RNA was added to each reaction. And run at the following the temperature: 25°C for 10 min, 42°C for 1 h, 85°C for 5 min.

### 2.15 Real time qPCR

cDNA was diluted 1:20 in RNAse free water for use in reactions. Quantitect primer assays (Qiagen) listed in Supplementary Table S1 with power SYBR green master mix (Thermo Fisher) were used for qPCR. qPCR was run on QuantStudio 7 Flex (Thermo Fisher). PCR cycle parameters were: 10 min at 95°C initial incubation followed by 15 s at 95°C and 1 min at 60°C cycles. Each run consisted of 40 cycles. After these cycles a melt curve ramp was run. Data was normalized to the mean expression of 18 s, HPRT1 and RPLP0 to obtain ${\Delta C}_{T}$ value. $-2^{\Delta \Delta C_{T}}$ was calculated and used for all subsequent analysis.

### 2.16 Statistical analysis

All statistical analysis was performed using GraphPad Prism 10.0 (San Diego, CA, United States) or SigmaPlot 12 (Grafiti, Palo Alto, CA, United States). All data are expressed as mean ± SEM. Significant differences were identified with unpaired *t*-test, two-way ANOVA, paired *t*-test, Wilcoxon signed rank test, or one-sample signed rank test. The difference was assumed to be significant at *p* ≤ 0.05.

## 3 Results

### 3.1 The gross anatomical differences in both sexes

**Figure 1:**
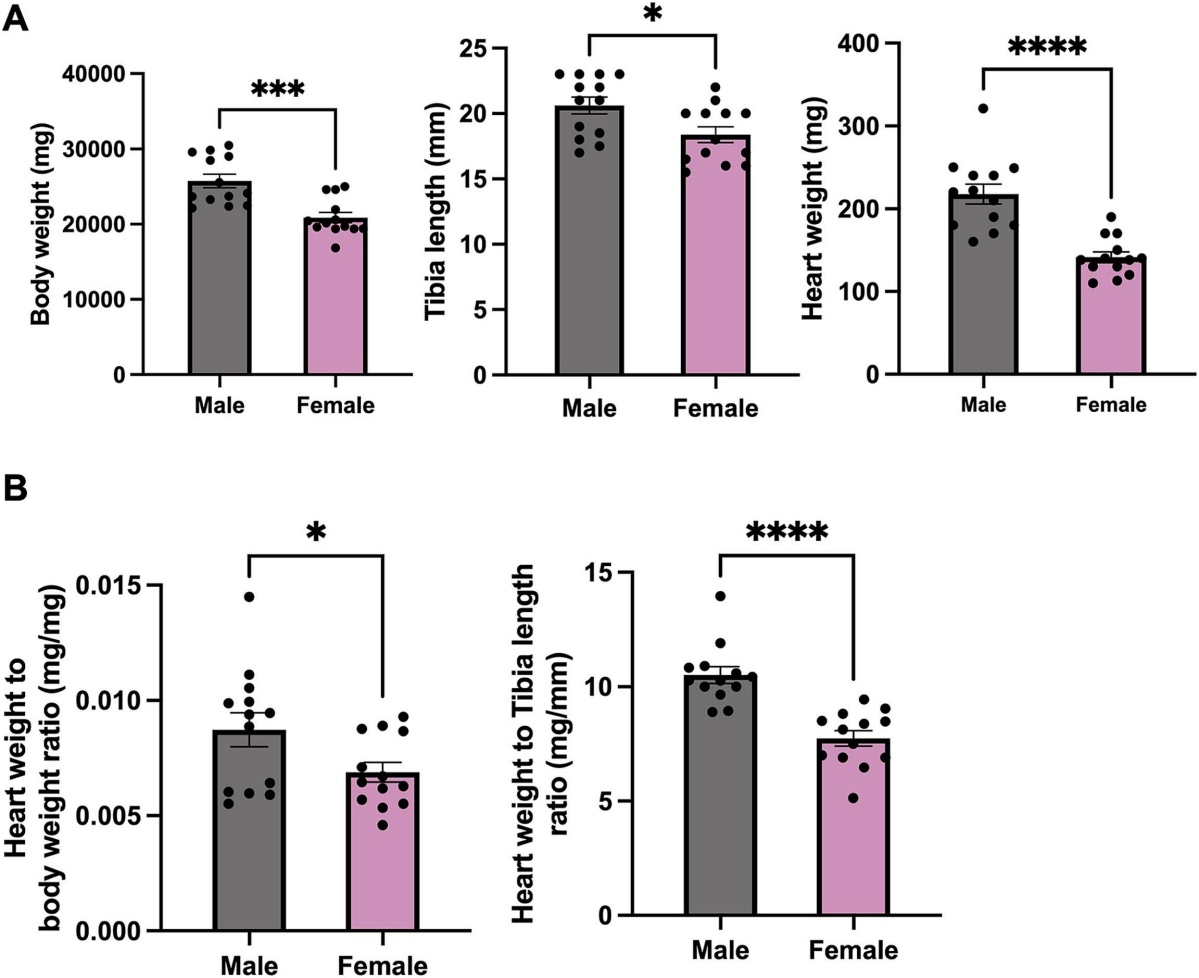
Phenotype data for male and female mice. **(A)** Body weight, heart weight and tibia length of female and male mice. **(B)** Heart weight to body weight and to tibia length ratio of female and male mice. *N* = 13. All values are shown as Mean ± SEM. ^*^p < 0.05, ^***^p < 0.001, and ^****^p < 0.0001. Unpaired *t*-test was used to analyse the data between two groups.

To primarily understand the gross anatomical differences in both sexes, body weight, heart weight and tibia length were measured. Significant differences of phenotype between male and female mice in terms of body weight, heart weight and tibia length were observed (Figure 1A), with male mice exhibiting significantly higher body and heart weight, as well as longer tibia length. The ratio of heart weight to body weight and heart weight to tibia length was notably higher in male mice compared to that in female mice (Figure 1B).

**Figure 2:**
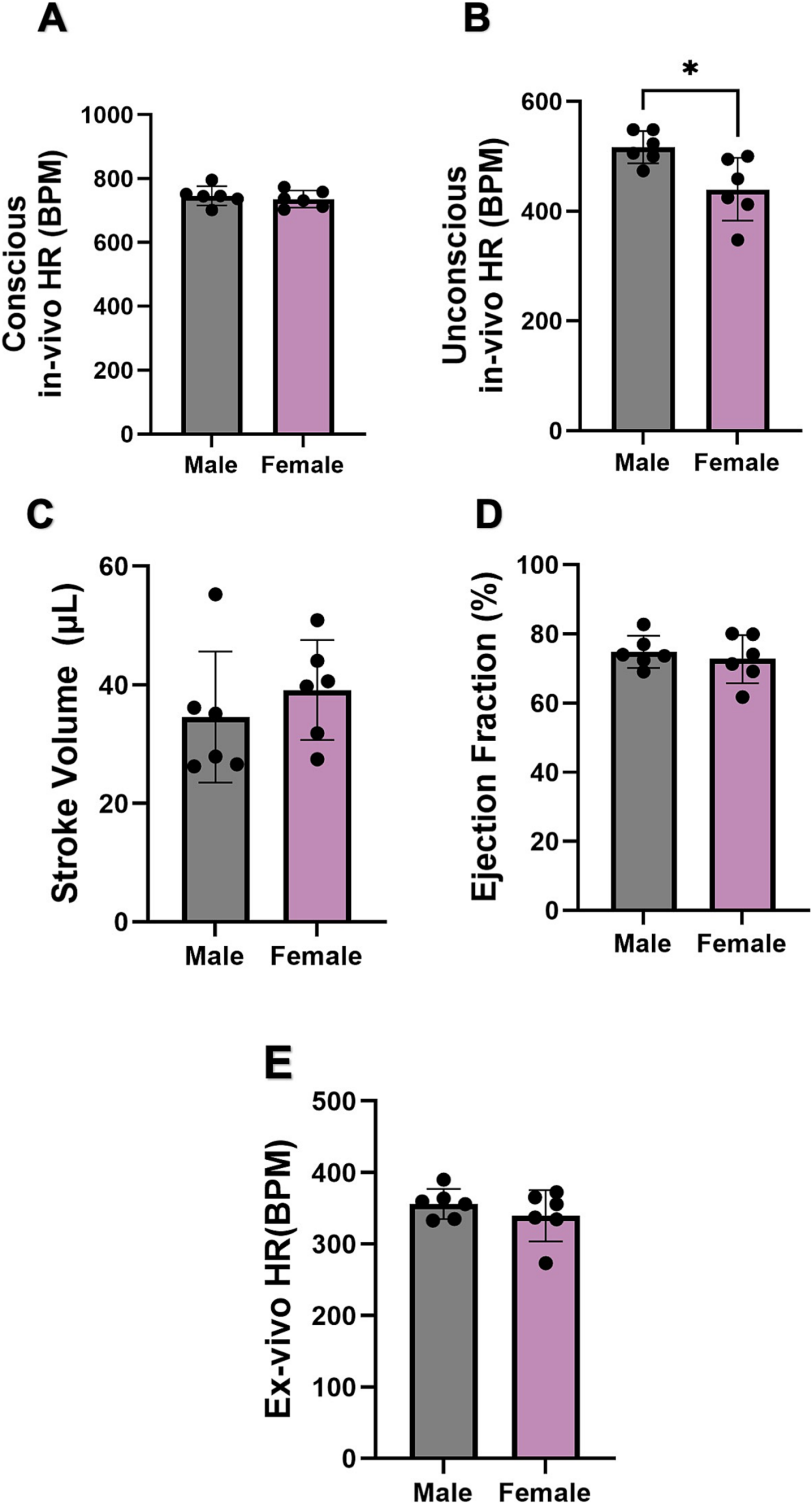
Comparison of cardiac function between male and female mice. **(A,B)** Comparison of conscious *in-vivo* HR and unconscious *in-vivo* HR between male and female mice recorded by ECG and echocardiography. *N* = 6 for males and *N* = 7 for females. **(C,D)** Comparison of stroke volume and ejection fraction between male and female mice via echography. **(E)** Comparison of *ex-vivo* HR between male and female mice. All values are shown as mean ± SEM. ^*^p < 0.05. Unpaired *t*-test was used to analyse the data between two groups.

The cardiac function, including *in*- and *ex-vivo* HR, SV, and EF, of both sexes were further explored. There was no difference when measuring *in-vivo* HR of conscious mice (Figure 2A). On the other hand, the *in-vivo* HR of unconscious female mice was significantly lower than that of males (Figure 2B). However, the SV, EF, and *ex-vivo* HR (measured in Langendorff-perfused hearts) showed no difference between sexes (Figures 2C–E).

**Figure 3:**
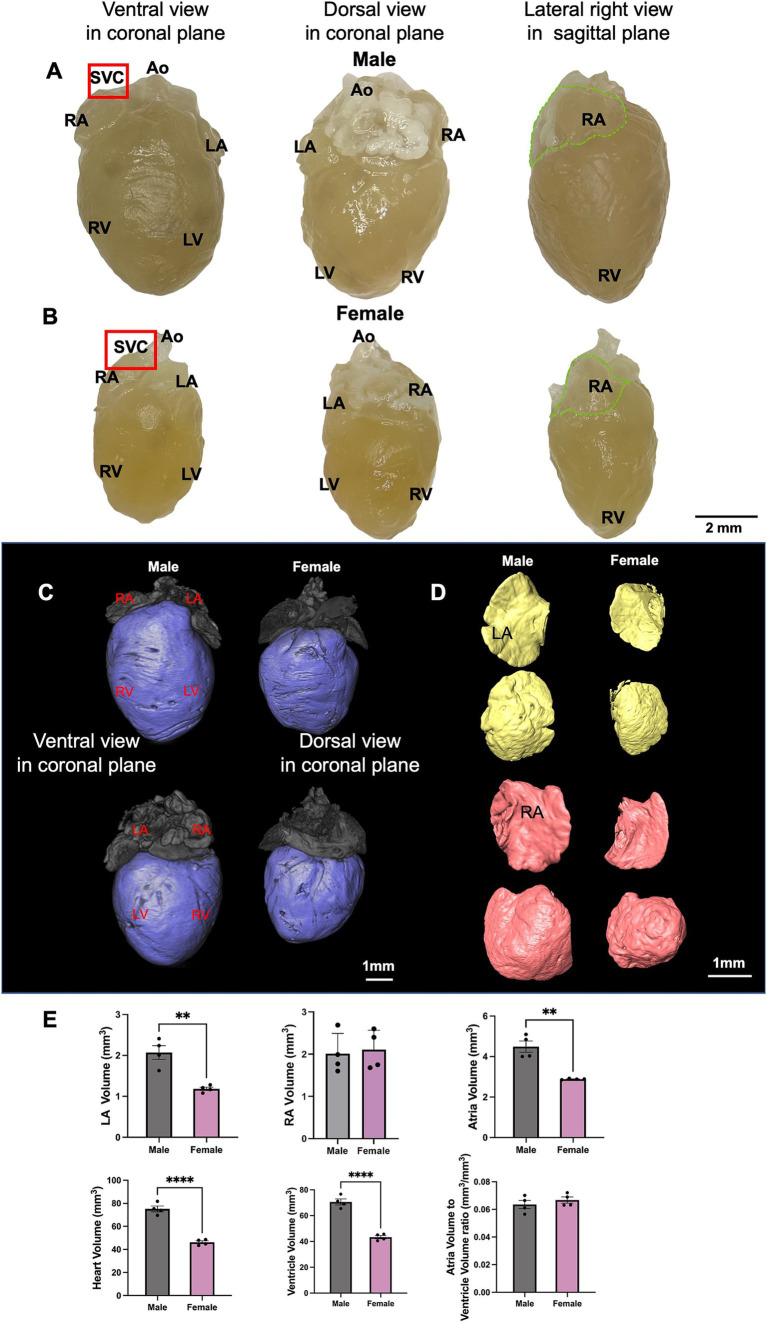
The anatomy of male and female mice heart. **(A,B)** Different aspects of the male and female mouse heart including ventral view, dorsal view and lateral right view are shown. The area where the SN is located is indicated by the red box and RA is marked by the dotted green line. **(C,D)** An illustration of segmented ventricle (blue), RA (pink) and LA (yellow) of young male and female mice from ventral and dorsal views are shown. **(E)** LA, RA, both atria, ventricular (LV, RV and septum), whole heart, and atria/ventricular volume ratio in male and female mice are shown. *N* = 4 for males and *N* = 4 for females. All values are shown as mean ± SEM. ^**^p < 0.01 and ^****^p < 0.0001. Unpaired *t*-test was used to analyse the data between two groups. Ao, aorta; LA, left atrium; LV, left ventricle; RA, right atrium; RV, right ventricle; SVC, superior vena cave.

The gross anatomical difference between the size of the male and female hearts can be seen (Figures 3A,B). Quantification of the ventricular and right atrial myocardial segmentation from the micro-CT data (Figures 3C,D) showed male mice exhibited a significantly larger LA, RA, ventricles, and heart volume than females (Figure 3E). This is consistent with the higher body weight in male mice compared to females. However, when we compared the ratio of atrial to ventricular volume, no significant difference was seen (Figure 3E).

### 3.2 SN microanatomy and expression of HCN channels in male and female mice

**Figure 4:**
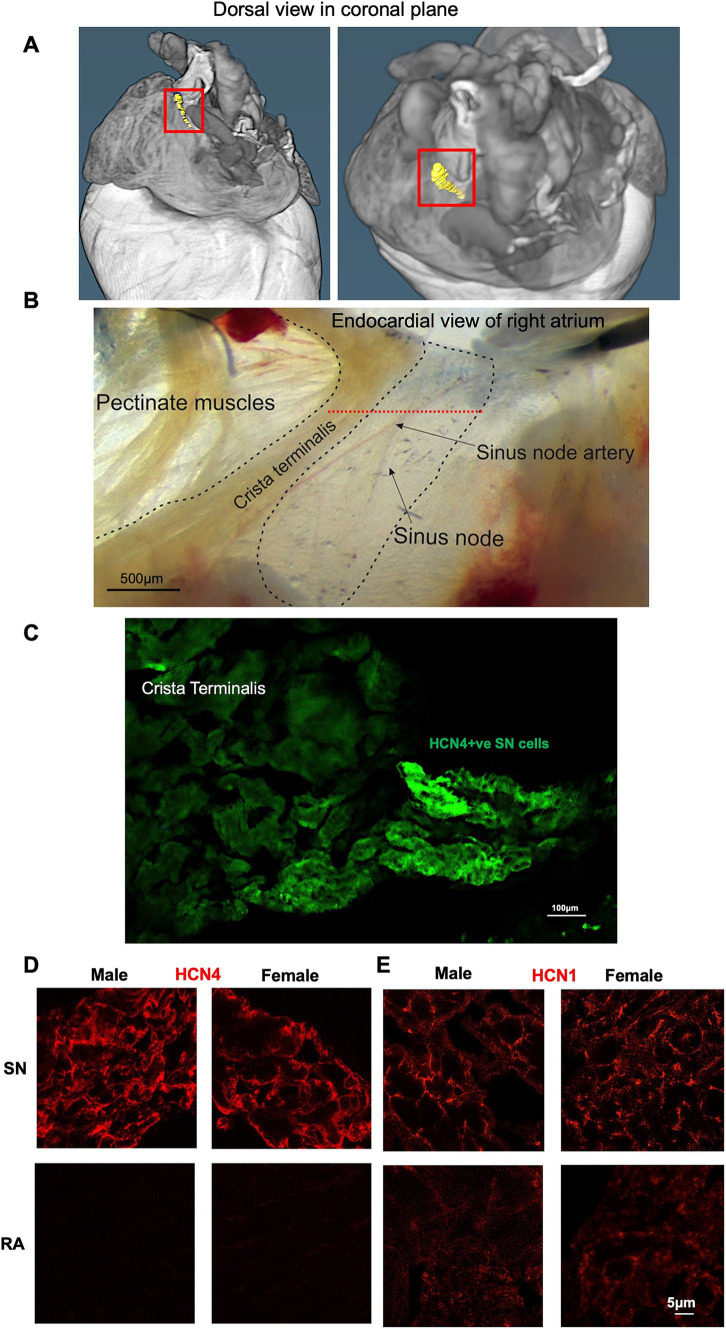
Immunohistochemical characterization of SN and RA in male and female mice. **(A)** Segmentation of the SN (shown in yellow and framed with the red box). **(B)** Example of SN/RA preparation with the SN region outlined. Level of leading pacemaker site shown by red dotted line, determined by sharp microelectrode measurement. **(C)** Immunohistochemistry targeting HCN4 within one SN/RA tissue section. **(D)** Immunohistochemistry targeting HCN4 within the SN and RA area of male and female mice. **(E)** Immunohistochemistry targeting HCN1 within the SN and RA area of male and female mice. RA, right atrium; SN, sinus node.

Micro-CT imaging was used to recognize the specific location of SN in mice, while immunofluorescence techniques were applied to evaluate the expression levels of HCN1 and HCN4 proteins in semi-quantitative way. Through micro-CT analysis, the SN was segmented (Figure 4A). Upon isolation of the SN/RA tissue, the SN tissue, depicted in Figure 4B, the high abundance of HCN4 protein in the SN was shown with strong positive immune-labelling (Figure 4C).

**Figure 5:**
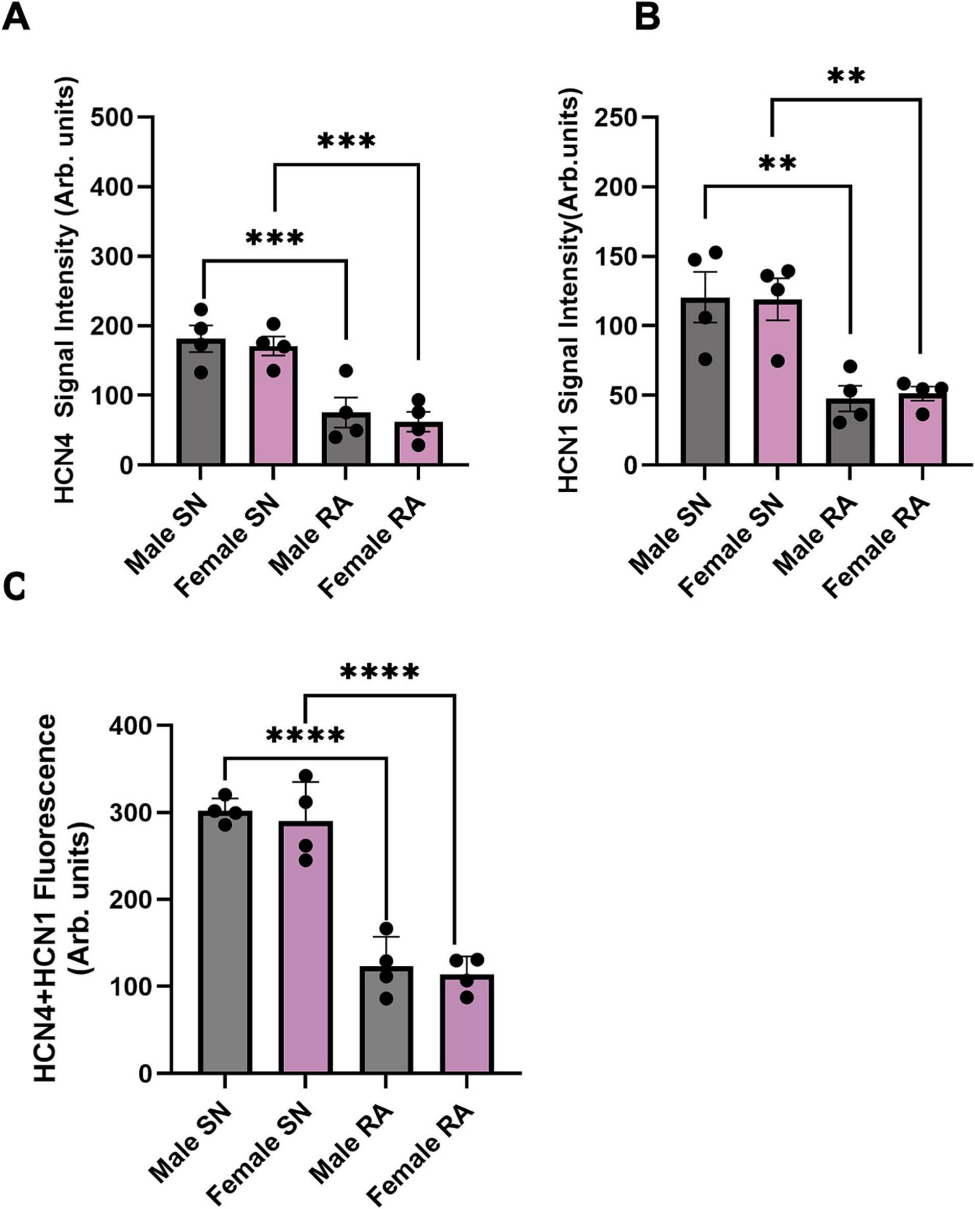
Semi-quantitative analysis of HCN4 and HCN1 signal intensity in SN and RA in male and female mice. **(A)** Semi-quantitative analysis of HCN4 between SN and RA in male and female mice. **(B)** Semi-quantitative analysis of HCN1 between SN and RA in male and female mice. **(C)** Semi-quantitative analysis of HCN4 and HCN1 between SN and RA in male and female mice. *N* = 4 for each. All values were shown as mean ± SEM. ^**^p < 0.01, ^***^p < 0.001, and ^****^p < 0.0001. Unpaired *t*-test was used to analyse the data between two groups. RA, right atrium; SN, sinus node.

Further investigations involved staining of consecutive tissue sections, which were sliced perpendicular to the CT, revealing membrane labelling for HCN1 and HCN4 proteins in the SN from both male and female mice (Figure 4C). HCN4 immunolabeling was poorly detectable in the RA, while detectable background signal for HCN1 was observed in the RA in both males and females (Figures 4D,E). Semi-quantitative analysis of the signal intensity confirmed these observations, showing a significantly higher expression of HCN4 and HCN1 in the SN compared to the RA (Figures 5A,B). The combined levels of HCN1 and HCN4 were also greater in the SN than in the RA (Figure 5C). We did not observe any significant differences in the signal intensities of HCN1, HCN4, or the combined signals of HCN1 and HCN4 in the SN between sexes (Figures 5A–C).

### 3.3 *I*_f_ in SN cells from male and female mice

To investigate sex differences and similarities in expression and gating of *I*_f_ across both sexes, *I*_f_ was recorded in SN cells isolated from SN tissue from either sex. We tested potential sex differences in the sensitivity of *I*_f_ to ISO by recording currents in the same cells perfused first with 1 nM ISO as a basal condition and then following wash-on of 1 μM ISO, to saturate cAMP synthesis and record *I*_f_ at maximum positive shift of activation curve. 1 nM ISO was used as an appropriate control condition as in previous studies (5, 9, 10) because it recapitulates resting catecholamine levels in mammals (41–43) and stabilizes the AP firing rate by eliminating frequent pauses and allowing longer recordings (10, 44). Notably, 1 nM ISO does not change the firing rate from that observed during the bursts of APs in the absence of ISO (no ISO: FR = 313 ± 31 AP/min, *n* = 19; 1 nM ISO: FR = 304 ± 15 AP/min, *n* = 50) (9) or the voltage-dependence of activation of *I*_f_ (no ISO: *V*_1/2_ = −104.3 ± 1.5, *n* = 16; 1 nM ISO: *V*_1/2_ = −103.7 ± 2.1, *n* = 20) (45).

**Figure 6:**
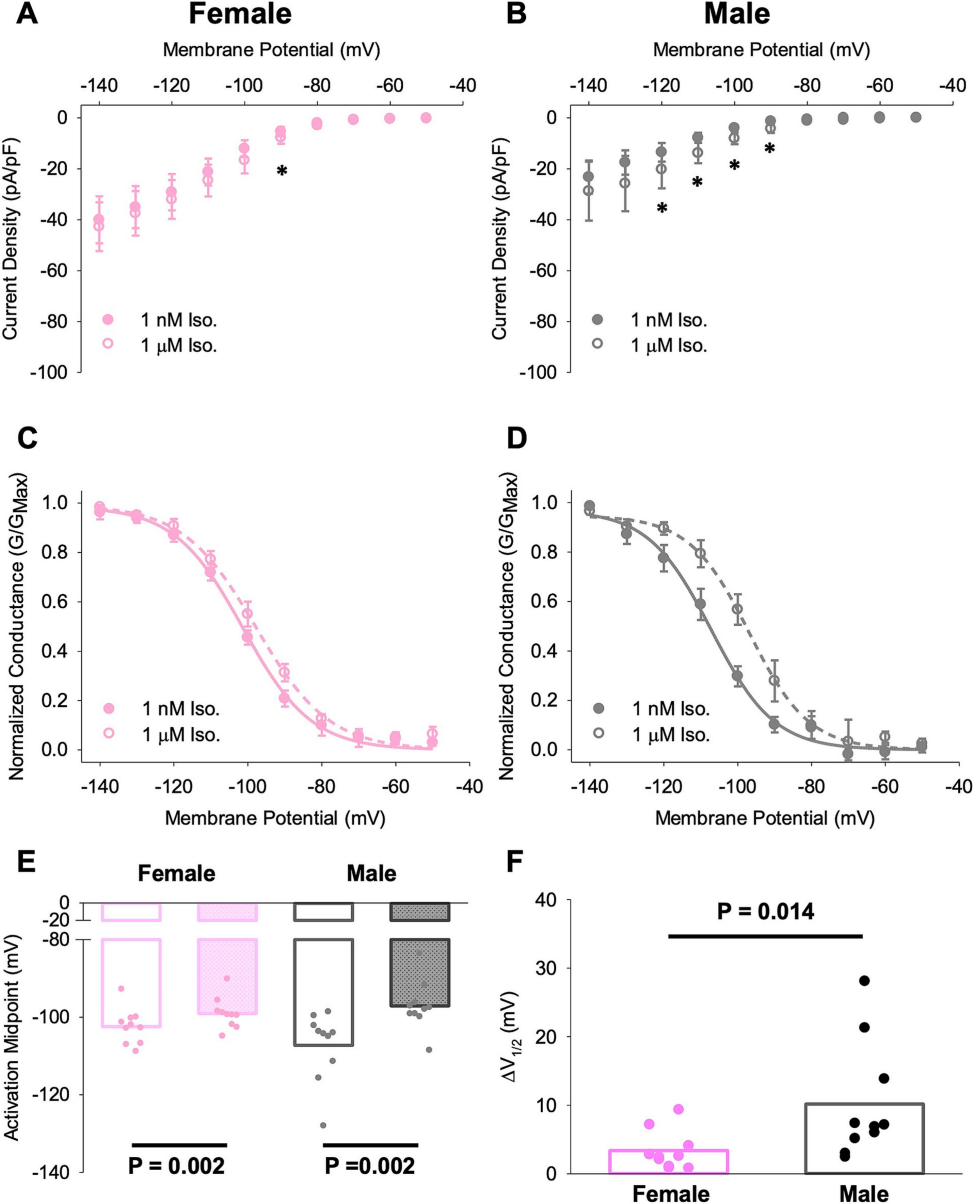
Different responses of SN cells from male and female mice to ISO treatment. **(A,B)** Current–voltage relationship of *I*_f_ in female **(A)** and male **(B)** SN cells in 1 nM and 1 μM ISO. Asterisks indicate *p* < 0.05 for the comparison between current densities at the indicated potentials in 1 nM and 1 μM ISO (paired *t*-test). There were no significant differences in current density between cells from male and female animals. **(C,D)** Voltage dependence of activation for *I*_f_ in female **(C)** and male **(D)** SN cells in 1 nM and 1 μM ISO. **(E)** Half-activation voltages (*V*_0.5_) for *I*_f_ in SN cells in 1 nM and 1 μM ISO. *N* = 3, *n* = 10 (female); *N* = 4, *n* = 10 (male). **(F)** ISO-dependent shifts in the midpoint activation voltage for *I*_f_ in individual cells compared by paired *t*-tests. All values are shown as mean ± SEM. SN, sinus node.

We found that wash-on of 1 μM ISO produced a significant increase in current density at some potentials near the midpoint activation voltage in SN cells from both males and females (Figures 6A,B). Correspondingly, ISO produced significant depolarizing shifts in *I*_f_ activation curve in cells from both female and male animals (Figures 6C–E). However the shift in activation midpoint (*V*_0.5_) was significantly larger in individual SN cells from males compared to cells from females (^*^p < 0.05, Figure 6F).

### 3.4 Sex disparities in spontaneous APs

Sharp microelectrode was employed in cSN and pSN to investigate differences of AP characteristics. The recordings were obtained from spontaneously beating preparations. The spontaneously beating rates in ms were: male cSN 121 ± 6.4 (raged 104–151); male pSN 121 ± 4.4 (range 106–123); female cSN 112 ± 4.1 (range 84–153); female pSN 112 ± 3.1 (range 97–133).

**Figure 7:**
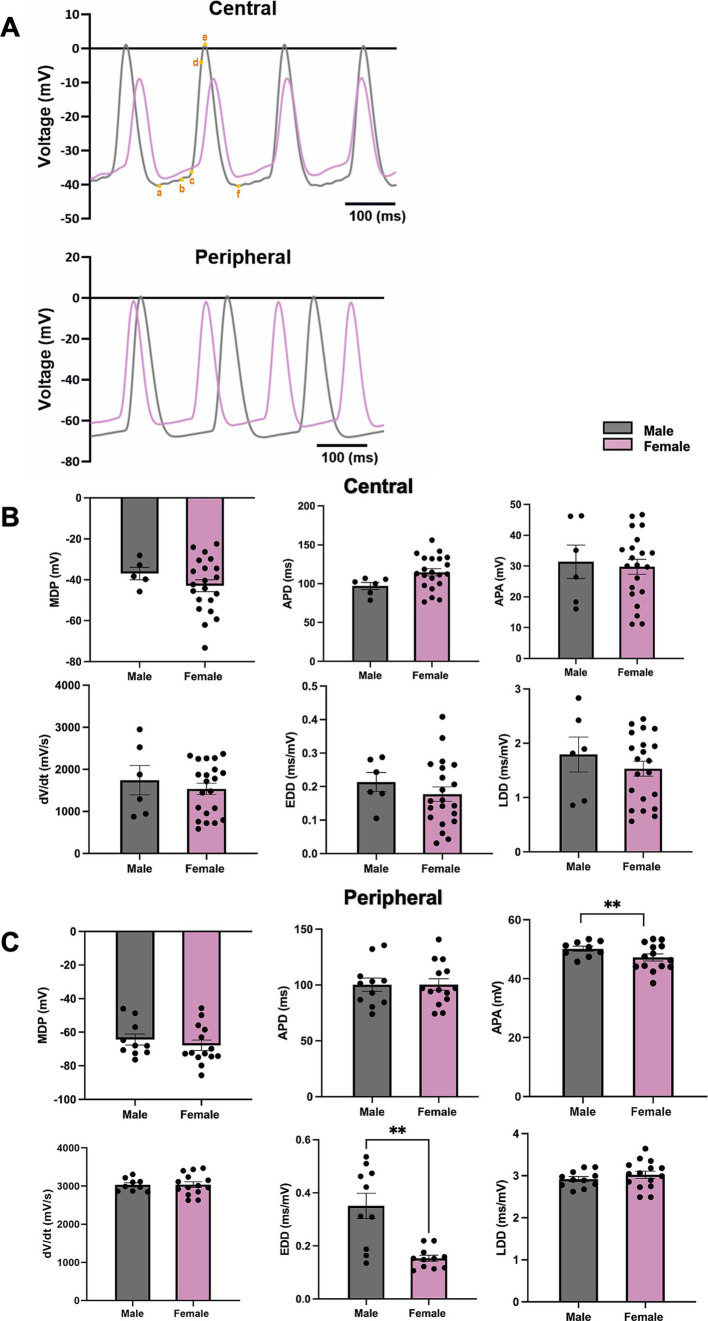
The measurements of AP parameters in cSN and pSN from male and female mice. **(A)** Representative AP from male and female SN (a,b, EDD; b,c, LDD; c,d, d*V*/d*t*; b–f, APD; a–e, APA; a, MDP). **(B)** AP parameters including MDP, APD, APA, d*V*/d*t*, EDD, LDD from cSN of male and female mice. *N* = 3, *n* = 6 (male); *N* = 4, *n* = 21 (female). **(C)** AP parameters including MDP, APD, APA, d*V*/d*t*, EDD, LDD from pSN of male and female mice. *N* = 4, *n* = 10 (male) and *n* = 14 (female). All values are shown as mean ± SEM. ^**^p < 0.01. Unpaired *t*-test was used to analyse the data between two groups. APD, action potential duration; APA, d*V*/d*t*, upstroke velocity; EDD, early diastolic depolarization rate; LDD, late diastolic depolarization rate; MDP, maximum diastolic depolarization.

The representative APs of cSN (top) and pSN (bottom) from male and female mice are shown in Figure 7A. In pSN tissue, the action potential amplitude (APA) and the slope of the exponential fraction of diastolic depolarisation (EDD) from male mice were significantly higher than these from female mice, while the maximum diastolic potential (MDP), the action potential duration (APD), the AP upstroke velocity (d*V*/d*t*), and the slope of the linear fraction of diastolic depolarisation (LDD) showed no difference (Figure 7C). In cSN cells, there was no sex difference found in these parameters (Figure 7B).

### 3.5 Mapping molecular architecture of the SN in male and female mice

**Figure 8:**
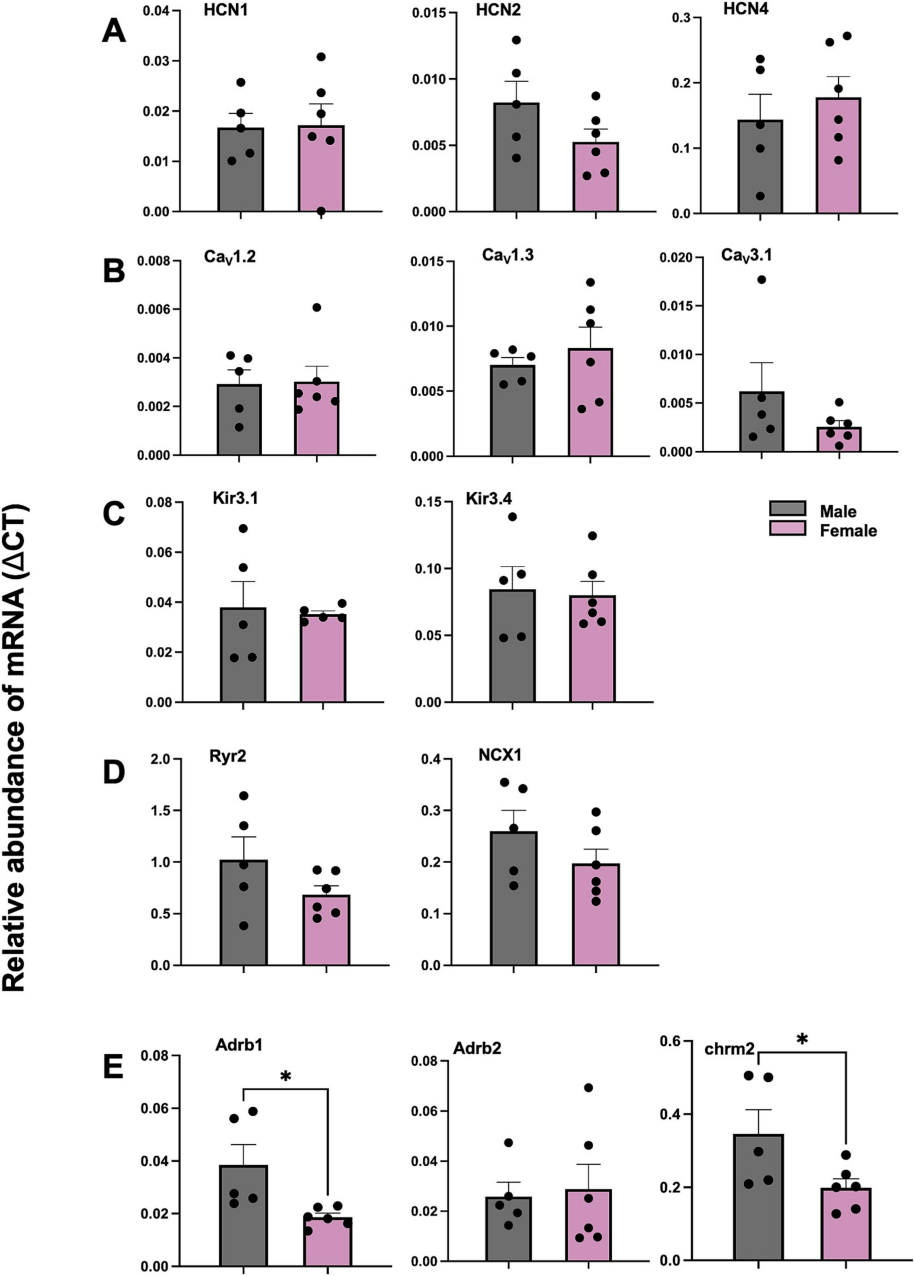
Comparison of mRNA for SN from male and female mice via qPCR for HCN, Ca^2+^, K^+^ channel subunits, calcium handling proteins, adrenergic receptors and cholinergic receptor. **(A)** Abundance of mRNA for HCN channel subunits in SN from male and female mice. **(B)** Abundance of mRNA for Ca^2+^ channel subunits in SN from male and female mice. **(C)** Abundance of mRNA for K^+^ channel subunits in SN from male and female mice. **(D)** Abundance of mRNA for calcium handling proteins mRNA in SN from male and female mice. **(E)** Abundance of mRNA for adrenergic and cholinergic receptor in SN from male and female mice. $\Delta C_{T}$ values shown as mean ± SEM. *N* = 5 (male); *N* = 6 (female). ^*^p < 0.05. SN sinus node.

The mRNA expression level of adrenergic receptor beta 1 (Adrb1) was significantly higher in male versus female mice (^*^p < 0.05, Figure 8E). The mRNA expression level of muscarinic acetylcholine receptor M2 (chrm2) was also significantly higher in male versus female mice (^*^p < 0.05, Figure 8E). However, other mRNAs—HCN, Ca^2+^, K^+^ channel subunits including HCN1, HCN2, HCN4, CACNA1C (Ca_V_1.2), CACNA1D (Ca_V_1.3), KCNJ3 (K_ir_3.1), KCNJ5 (K_ir_3.4), Ryr2, NCX1, as well as Adrb2, showed no difference between female and male mice (Figures 8A–D). CACNA1G (Ca_V_3.1) mRNA showed trend to higher expression in males than females (Figure 8B), which may contribute to faster EDD in the periphery of the SN (Figure 7C).

### 3.6 Sex disparities in SN automaticity and distribution of pacemaker leading sites in SN tissue from male and female mice

**Figure 9:**
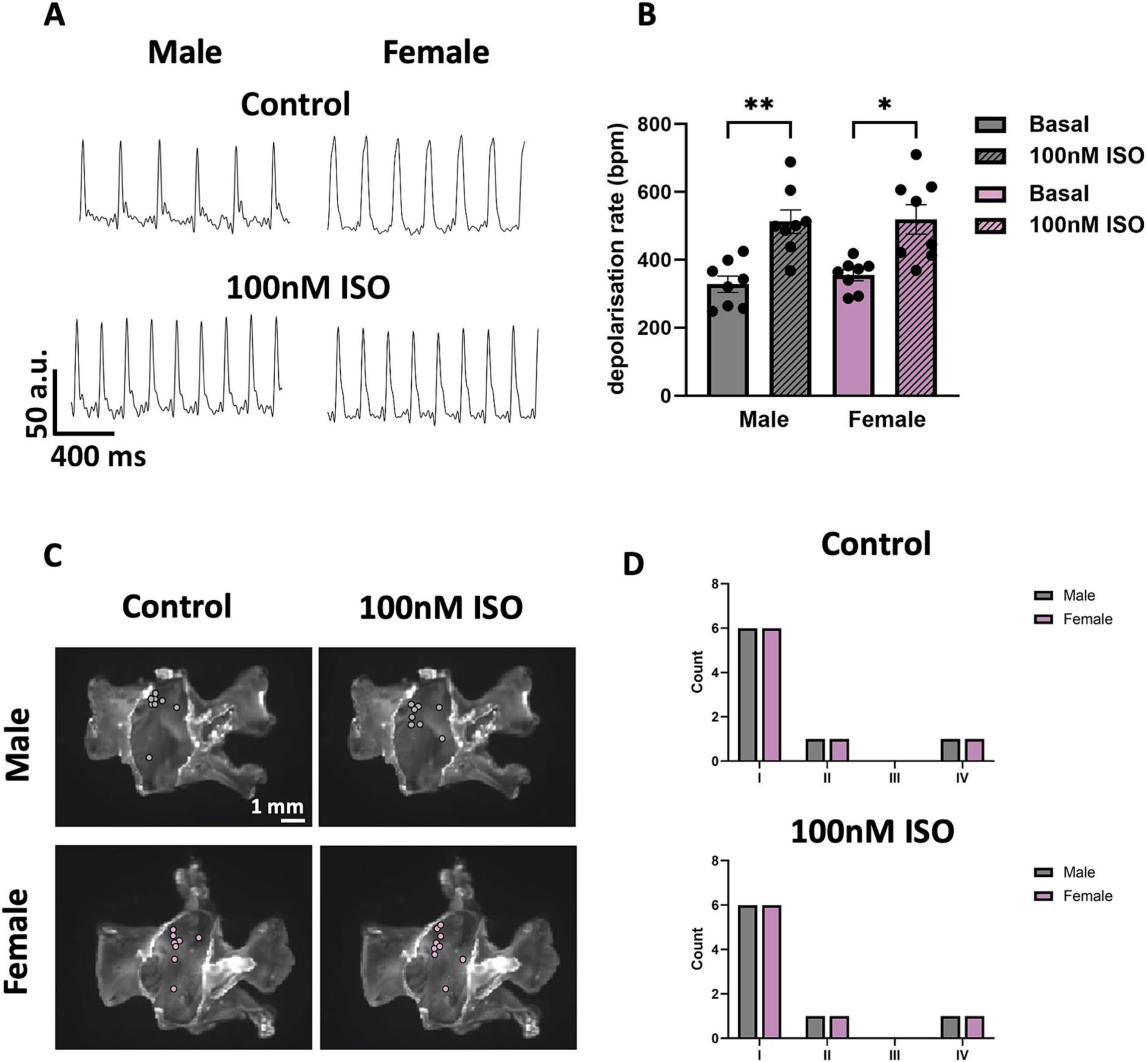
Automaticity and distribution of pacemaker LR in SN tissue from male and female mice. **(A)** Representative traces of voltage signal from male and female SN tissue without or with 100 nM ISO. **(B)** Comparison between depolarisation rates from male and female SN tissue without or with 100 nM ISO. **(C)** Sample snapshots of SN/RA tissues with points showing the position of the pacemaking LR. **(D)** Counting of the LRs into four categories depending on the alpha angle value calculated. *N* = 8 (male); *N* = 8 (female). All values are shown as mean ± SEM. ^*^p < 0.05 and ^**^p < 0.01. LR, leading region.

Mice of both sexes showed similar frequency of spontaneous SN APs at baseline, and similar positive chronotropic response to superfusion of ISO 100 nM (Figures 9A,B). The spatial distribution of pacemaker leading regions did not show significant differences in either sex at baseline or in ISO (Figures 9C,D). The percentage of preparations displaying the legion region in the different angle categories is 75% (I) versus 25% (II, IV), in both control and ISO 100 nM conditions (see Methods).

## 4 Discussion

Understanding sex differences in cardiac electrophysiology and structure-function relationships is critical due to the distinct physiological and pathological characteristics observed between males and females. These differences encompass variations in myocardial size, action potential dynamics, ion channel expression, and conduction properties (46–49), all of which contribute to differential responses to cardiac stressors. For instance, females often exhibit increased susceptibility to conditions such as heart failure with preserved ejection fraction (HFpEF) and specific arrhythmias, partly due to their distinct myocardial stiffness, hormonal influences, and autonomic regulation (50, 51). On the other hand, males are more prone to fibrosis and structural remodelling, factors that exacerbate the risk of arrhythmias in larger hearts (52). Investigating these sex-based variations is essential for developing more precise diagnostic tools and therapies, as they play a pivotal role in shaping the clinical presentation, progression, and outcomes of cardiac diseases. Furthermore, the interplay between electrophysiological properties and structural remodelling in males and females calls for a deeper exploration of how these differences impact arrhythmogenesis, response to pharmacological interventions, and long-term prognosis. As we advance our understanding of these mechanisms, incorporating sex-specific data will be essential for tailoring treatment strategies that are not only more effective but also equitable across sexes, thus addressing the current gaps in cardiac care.

### 4.1 Sex differences in heart volume

In our study, the heart volumes, including the LA, whole atria, whole ventricles, and whole heart, were significantly larger in male mice compared to females. The relationship between myocardial volume and electrical propagation, including the susceptibility to arrhythmias, delves into the intricate interplay between heart size and its electrical properties (53). In this case, males with larger hearts, due to their increased myocardial tissue, might be predisposed to a higher incidence of arrhythmias, a phenomenon not as prevalent in smaller hearts, such as those of female mice. Increased heterogeneity in electrical conduction, variations in gap junction distribution, ion channel expression, and larger heart size with associated fibrosis contribute to a heightened susceptibility to arrhythmias such as atrial fibrillation in larger hearts, as demonstrated by both comparative physiology studies and computational models (53–58). HFpEF involves stiffened myocardial tissue that impairs efficient filling, commonly associated with hypertrophy, larger heart size, atrial enlargement, and fibrosis, which contribute to arrhythmias and complicate clinical management due to both structural and electrical abnormalities (59–63). More importantly, Mesquita et al. (63) has shown intrinsic abnormalities of SN structure and function underlie the chronotropic response in HFpEF. This suggests that males, who typically have larger hearts, may be more prone to HFpEF and even sinus node dysfunction (SND), which thus require heightened medical attention. This complex relationship underscores the importance of considering both the intrinsic electrical properties of cardiac tissue and the structural challenges posed by larger myocardial volumes in understanding and addressing arrhythmogenesis in larger mammalian hearts.

### 4.2 Influence of sex on SN

The ability of the SN to generate spontaneous APs required for cardiac pacemaking is dependent on the expression of key membrane bound ion channels including HCN and calcium channels (64). We found similar HCN1 and HCN4 levels in the male and female SN, paralleling our earlier study (31). Although there is no significant difference in conscious *in-vivo* HR or *ex-vivo* HR, unconscious *in-vivo* HR in male mice manifested 17.5% higher compared to their female counterparts, which could be explained by the differential sensitivity of autonomic input to sedation in males and females. In addition, it is possible that HR in females is more sensitive to sedation because of female specific hormonal factors.

HCN1 and HCN4 are crucial components in the physiology of the SN. HCN channels, including HCN1 and HCN4, are integral to SN function. These channel isoforms contribute to generation of native SN f-channels and *I*_f_, which is pivotal in setting and regulating the pacemaker activity (65, 66). Whilst we did not see any sex-related difference in the expression of HCN1 or HCN4, the differences from *in-vivo* HR may be due to sex-related differences in the expression of other ion channels involved in SN pacemaking, such as Ca_V_1.3 or Ca_V_3.1 Ca^2+^ channels or may be related to the atrial myocardial volume differences that we observed. Mangoni et al. (17) demonstrated that Ca_V_1.3/L-type Ca^2+^ channels play a major role in the generation of cardiac pacemaker activity by contributing to DD in SN cells using Ca_V_1.3 knockout mice. Similarly, Mangoni et al. (67) showed that Ca_V_3.1/T-Type Ca^2+^ channels contribute to SN pacemaker activity by utilizing Ca_V_3.1 knockout mice. Thus, the molecular architecture of the SN in sex differences was investigated. Although HCN, Ca^2+^, K^+^ channel subunits showed no difference in mRNA level in both sexes of mice, our previous paper showed that the mRNA and protein level of CACNA1D (Ca_V_1.3) are differentially expressed in female than male in rats (31). Therefore, the proteins for calcium channels and calcium handling proteins should be further explored in mice for both sexes that could potentially explain the observed difference in *in-vivo* HR.

Interestingly, the observation that the mRNA level of Adrb1 and Chrm2 (M2 muscarinic receptor) are significantly lower in females than in males provides valuable insights into sex-specific differences in SN function and responses to stress. Adrb1 is crucial for the sympathetic regulation of the heart, influencing HR, myocardial contractility, and overall cardiac output. The lower expression of Adrb1 in females might suggest a reduced cardiac response to catecholamines such as adrenaline, which is pivotal during acute stress responses (68, 69). This reduction could lead to a lower likelihood of rapid HR and contractility increases in females compared to males. This is particularly relevant given that lower expression of Adrb1 in females may offer some protection against catecholamine-induced cardiotoxicity in SN.

The lower expression of Chrm2 in females also adds an important layer to understanding sex-specific differences in SN function. Chrm2 is primarily involved in mediating parasympathetic (vagal) control over the heart (70), which slows the HR and increases HRV. A meta-analysis has shown that HRV in women is characterized by a relative dominance of vagal activity despite greater HR (71). With reduced Chrm2 expression, females may experience less parasympathetic influence on the heart. This could mean a reduced capacity to slow down the heart rate quickly after stress or during rest, leading to a narrower range of HRV (72). While this might initially seem disadvantageous, it could contribute to a more consistent heart rate pattern, reducing the extremes of high and low heart rates.

Combining the lower expression of both Adrb1 and Chrm2 in females suggests a unique autonomic balance: less sympathetic (due to lower Adrb1) and parasympathetic (due to lower Chrm2) responsiveness. This balance might result in a more stable and less reactive HRV profile in females, which could protect against the potential negative effects of both excessive sympathetic activation (like arrhythmias) and extreme parasympathetic influence (like bradycardia). Therefore, the sex-specific expression patterns of these receptors could be key factors in the different cardiovascular responses to stress and the overall autonomic regulation of SN function in males and females.

Moreover, as 1.5% isoflurane anesthesia was used, the observed differences in the autonomic input to sedation between sexes may be attributed to the lower expression levels of key receptors (Adrb1 and Chrm2) involved in autonomic regulation (73, 74). Specifically, Adrb1 is a key receptor for sympathetic stimulation, which increases heart rate (75, 76). With lower levels of Adrb1, females may inherently have a reduced sympathetic response. Under sedation, which already dampens sympathetic activity, this lower baseline responsiveness in females may lead to an even more pronounced decrease in heart rate compared to males (77–79). Moreover, the reduced expression of Chrm2 (M2 muscarinic receptor) in females could exacerbate the effects of sedation on HR by further reducing parasympathetic input, leading to a greater bradycardic response (80). These findings suggest that sex-specific receptor expression profiles could underlie the differential sensitivity to sedation observed in females, warranting further investigation into the interplay between autonomic regulation and sex differences in heart rate control. In another study it was shown that females have 20–30% greater sensitivity to the muscle relaxant effects of vecuronium, pancuronium and rocuronium (81). Females are more sensitive than males to opioid receptor agonists, as shown for morphine as well as for a number of kappa (OP2) receptor agonists (81). For opioid analgesics, females may experience respiratory depression and other adverse effects more easily if they are given the same doses as males (81). Together, these observations underscore the importance of considering sex-specific responses in anesthesia and analgesia to optimize treatment protocols and minimize adverse effects across sexes.

In parallel with the observation of lower Adrb1 expression in female SN compared to male, we found a reduced response to ISO in female SN cells compared to male SN cells using 1 nM ISO as the control condition and wash-on of 1 M ISO as the stimulated condition (Figure 6). We have previously reported that, at least for SN cells for males, 1 nM ISO does not have any effect on either the voltage-dependence of activation of *I*_f_ or on the AP firing rate; rather it is used to eliminate the pauses in spontaneous APs that are common in the absence of ISO (5, 9, 40). Moreover, in cells from males, we have previously found that 1 μM ISO is required to achieve a full chronotropic response. The larger response to1 μM ISO in cells from males may be associated with the higher Adrb1 expression in males. The potential functional impact of these findings is significant, which could explain sex-based HR differences with ANS signalling. Although there is no difference in APs from central SN, we did observe a difference in EDD in APs from pSN. This discrepancy could be associated with higher expression of Adrb1 in males compared to females and perhaps higher Ca_V_3.1 expression.

Thus, even though there are no differences in *I*_f_ density between male and female mice, *I*_f_ shifts significantly after high concentration ISO in male but not female. Further investigation is needed at the protein level for ion channels, especially Ca_V_1.3, Ca_V_3.1 as well as HCN1 to determine molecular substrates for observed electrophysiological differences.

### 4.3 Oestrogen’s influence on SN function: unravelling sex-specific mechanisms

The observed differences in this study might be underlined by the influence of sex hormones on the cardiovascular system. For example, oestrogen affects cardiac functions, including calcium homeostasis, through a combination of genomic and non-genomic mechanisms (82). These mechanisms influence the expression and activity of various calcium-handling proteins in cardiac myocytes. For genomic actions, oestrogen binds to oestrogen receptors (ERs) which are nuclear transcription factors. ERs exist in two main forms, ERα and ERβ, both of which are expressed in cardiac tissue (83). The role of oestrogen metabolites, such as 16α-OHE1, further complements this understanding. Yin et al. (33) revealed that 16α-OHE1, can attenuate myocardial ischemia–reperfusion injury of the heart. Such findings imply that oestrogen and its metabolites may contribute to sex differences observed in cardiac functionality. Although the potential impact of sex-related hormones on the direct function and protein expression within pacemaker cells of key proteins, such as HCN4, which is highly expressed in the SN vs. RA (84), is still uncertain, oestrogen can influence cAMP levels (85). The binding of oestrogen to either nuclear (Oestrogen Receptor alpha and beta) or membrane-bound (G-protein coupled estrogen receptor, GPER) (57, 58), can activate various signalling cascades, particularly through GPER activation, stimulating adenylyl cyclase activity, leading to an increase in cAMP levels, and protein kinase A activation in cardiomyocytes (86, 87).

In the context of the heart, oestrogen-induced cAMP is a crucial regulator of cardiac function (82, 85, 88, 89). Oestrogen significantly influences cardiac function in mice by modulating the cAMP-L-type Ca^2+^channel pathway in left ventricular apical myocytes (90), as evidenced by changes in intracellular cAMP levels, contraction and Ca^2+^ transient amplitudes, and gene expression related to cardiac contraction in female and ovariectomized mice treated with oestrogen (85). Oestrogen’s ability to modulate cAMP levels therefore might also influence *I*_f_, contributing to the sex differences observed in special physiological conditions such as pregnancy (91) and responses to certain cardiac drugs (73, 92, 93). Understanding how oestrogen influences cAMP in SN cells can have implications for the development of sex-specific treatments for SND. For example, drugs that target cAMP signalling pathways might have different efficacies or side effects in men and women due to the modulatory effects of oestrogen.

### 4.4 Other factors involved in SN function and clinical implications

The difference in *in-vivo* HR and level of Adrb1between male and female SN have profound clinical implications, especially in the context of cardiac arrhythmias and the therapeutic targeting of ion channels. Despite these differences, our study revealed no disparity in HCN1 and HCN4 expression, which imply f-channel subunit composition is unlikely to explain the differential responsiveness of *I*_f_ to isoproterenol in female and male pacemaker cells. This suggests that other factors, possibly upstream of HCN4 or in parallel pathways, contribute to the variations in SN function and arrhythmia susceptibility between the sexes. Among those, our finding that Adrb1 expression is higher in males’ SN may explain sex-specific differences in *I*_f_ regulation. In addition, other factors such as myocardial volume-related fibrosis (56, 57), expression and regulation of L-Type Ca^2+^ channels (31, 85), oestrogen’s influence (94, 95) as mentioned above, or innate immune system (96) may contribute to sex-specific differences in heart rate regulation.

Sex differences in the innate immune system, marked by variations in inflammatory mediator levels and modulated by sex hormones, can indirectly influence cardiac electrophysiology and contribute to the disparity in arrhythmia prevalence between males and females (96). Women, with a generally stronger immune response, may experience more profound immune-mediated changes in cardiac ionic currents and membrane potentials, potentially leading to faster HR (97). Conversely, chronic inflammation can prompt fibrosis, altering cardiac conduction pathways and slowing down HR (98–100). Additionally, immune responses can affect the ANS (101), further modulating HR. (102). These nuanced immune-related differences underscore the importance of considering sex in understanding cardiac electrophysiological responses and in developing tailored treatments for SN conditions. Moreover, Hulsmans et al. (103) reported that resident macrophages can couple to cardiomyocytes via Cx43 and accelerate their repolarization in AVN. Are there more resident macrophages and related connexins in females than males in SN? Further investigation is needed.

Thus, while ivabradine, a known HCN4 blocker, remains a crucial tool in HR regulation (104), its efficacy and side-effect profile might be influenced by sex-specific factors not directly related to HCN1 and HCN4. Additionally, the impact of current block on HR regulation and arrhythmia propensity needs to be assessed with a sex-specific lens. Studies like those by Bucchi et al. (105), which explore the pharmacological modulation of pacemaker currents, provide a foundation for understanding how these interventions might yield different outcomes based on sex. The interplay between oestrogen, cardiac ion channels, and response to pharmacological agents, as highlighted in research by Yang et al. (106), further underscores the importance of personalized medicine in cardiac care. The necessity for a sex-specific approach in both research and clinical management of cardiac disorders becomes evident, as does the importance of personalization in cardiac therapeutics. Further investigation is essential to elucidate the molecular mechanisms that drive these sex differences in SN behaviour, which will inform the development of more tailored treatments for arrhythmias that consider the patient’s sex as a fundamental factor.

## 5 Conclusion

In our pursuit to understand the mechanisms governing HR, it became evident that sex-specific nuances significantly influence SN function and the underlying electrophysiological mechanisms in mice. We have found faster *in-vivo* unconscious HR, faster EDD and higher Adrb1 expression in males than females mice, emphasizing the need for personalized therapeutic strategies in cardiovascular medicine, and underscoring the paramount importance of incorporating sex perspectives in cardiac research. These findings suggest a more nuanced understanding of HR regulation and its variability across sexes and physical conditioning. It also highlights the importance of considering sex-based differences and training impacts when evaluating cardiac health and designing treatments for arrhythmias or pacemaker diseases, which paves the way for future studies to delve deeper into the clinical implications and mechanisms behind these sex-based disparities in the SN.

### 5.1 Strength and limitation

Our conclusions are based on murine models and, while insightful, might not wholly translated to human physiology. Although the molecular architecture in sex differences was mapped, the intricate mechanistic pathways underlying the observed sex-based differences in unconscious *in-vivo* HR remain unclear in this study.

## Data Availability

The datasets presented in this study can be found in online repositories. The names of the repository/repositories and accession number(s) can be found in the article/Supplementary material.

## Footnotes

^1^ https://imagej.net
