# Supplementary material for: Identifying sex similarities and differences in structure and function of the sinoatrial node in the mouse heart
Source: Front Med (Lausanne). 2024 Dec 5;11:1488478. doi: 10.3389/fmed.2024.1488478 (PMC11655232; doi:10.3389/fmed.2024.1488478)
Supplement: Supplementary file 1 [file Table_1.DOCX]

Supplementary Table

Supplementary Table 1 Qiagen QuantiTect primer assays used for qPCR

| **Target mRNA** | **Qiagen QuantiTect primer assay number** |
| --- | --- |
| **Adrb1** | QT00258692 |
| **Adrb2** | QT00253967 |
| **Cav1.2** | QT00053480 |
| **Cav1.3** | QT00076657 |
| **Cav3.1** | QT01870057 |
| **chrm2** | QT00290297 |
| **HCN1** | QT00172130 |
| **HCN2** | QT00112294 |
| **HCN4** | QT00038108 |
| **Kir3.1** | QT00030380 |
| **Kir3.4** | QT00070406 |
| **Ryr2** | QT00018368 |
| **NCX2** | QT00028609 |
